# Nexus between organizational citizenship behavior and psychological wellbeing: emotional intelligence as a pathway

**DOI:** 10.3389/fpsyg.2024.1389253

**Published:** 2024-08-21

**Authors:** KDV Prasad, Shivoham Singh, Ved Srinivas, Rajesh Vaidya, Krishna Kant Dave

**Affiliations:** ^1^Research, Symbiosis Institute of Business Management, Hyderabad, India; ^2^Operations, Symbiosis International (Deemed University), Pune, India; ^3^Human Resources, Thiagarajar School of Management, Madurai, Tamil Nadu, India; ^4^Human Resources, Symbiosis Institute of Business Management, Nagpur, India; ^5^University Administration, Shri Venkateshwara University, Gajraula, Uttar Pradesh, India

**Keywords:** emotional intelligence, civic virtue, managing emotions, autonomy, altruism

## Abstract

**Aim/purpose:**

The aim of this study is to investigate the nexus between organizational citizenship behavior and psychological wellbeing and assess the moderating and mediating effects of emotional intelligence (EI) on the relationship betwem psychological wellbeing of IT-enabled Sector employees in Hyderabad.

**Design/methodology/approach:**

To measure the study variables of organizational citizenship behavior (OCB) and emotional intelligence (EI) on psychological wellbeing (PWB) data were gathered using a questionnaire. The mediating and moderating effects of emotional intelligence on the relationship between organizational citizenship behavior and psychological wellbeing was also assessed. The was reliable as indicated by the Cronbach's alpha coefficient statistic that between 0.79 to 0.91.

**Findings:**

Three hundred valid responses were considered for SEM analysis using AMOS, version 28. The model fit indices indicate excellent fit: CMIN/DF 2.788 CFI 0.935, IFI 0.937, TLI 0.921, NFI 0.923, RMSEA 0.054, SRMR 0.077 and PClose 0.092. The SEM analysis revealed that the impact of exogenous variables OCB and EI were statistically significant (p < 0.001) on endogenous variable psychological wellbeing of IT-enabled industry employees. Furthermore, EI partially mediates psychological wellbeing through the OCB of information technology employees. This empirical study also examined the moderating effects of EI on the psychological wellbeing of information technology-enabled employees through OCB. The slope analysis reveals that emotional intelligence strengthens the positive association between OCB and the PWB of IT-enabled sector employees. EI and OCB enhance PWB and employee performance.

**Research implications/limitations:**

The findings of this study have several important implications for organizations in the IT sector and can be used to develop strategies for promoting OCB and EI among employees. The structural relationships between PWB and OCB in the context of hotel employees and reported positive effects of OCB on hotel employees are well documented. The limitations are the data were collected from the Information Technology employees of Hyderabad Metro. There are some subjectivity and cultural issues which were elaborated at the end

**Contribution/Originality:**

This empirical study helps to clarify the relationship between organizational citizenship behavior, psychological wellbeing, and the mediator and moderator variable emotional intelligence. The study also comprehends the available literature and adds value to the existing theoretical knowledge and behavioral studies.

**JEL classification:**

M10 M12, M19.

## 1 Introduction

Organizational citizenship behavior (OCB) is a flexible and open individual behavior in which an employee performs or assists colleagues after completing his or her usual job Helping coworkers with tasks, providing constructive criticism and feedback, performing extra jobs when necessary, participating in organizational events and activities, treating others with courtesy and respect at work, and taking the initiative to identify opportunities to improve the company's reputation in the community are just a few examples of how OCB may manifest. Beyond an individual's formal job obligations, OCB includes behaviors that have a beneficial impact on the organization's performance, culture, and atmosphere.

Psychological wellbeing refers to an individual's overall subjective wellbeing and life satisfaction, which includes both emotional and cognitive functioning. It is a multidimensional concept that incorporates many areas of a person's life, such as self-esteem, positive affect, personal development, autonomy, pleasant relationships, and environmental mastery at work.

Today's firms are under constant pressure to generate results, be innovative, and adapt to the rapidly changing business landscape. To achieve these stated aims, employees must be motivated, involved, and dedicated to their jobs. In this context, OCB has gained importance in organizational psychology. The term OCB refers to voluntary activities that workers conduct outside of their job responsibilities but that benefit the company's overall operation. These actions include hand-holding co-workers, demonstrating empathy, offering assistance, proposing enhancements, taking part in the decision-making process, and remaining devoted to the company. Another crucial concept in psychology research that has attracted much attention is psychological wellbeing (PWB). It describes a person's subjective assessment of his or her general level of mental health and contentment in life. Positive affect, personal development, life purpose, environmental mastery, autonomy, and self-acceptance are among the elements that constitute PWB (Ryff and Keyes, 1995). Studies have indicated that workers with high PWB levels are happier, more productive, and more devoted to their companies (Chuang et al., 2024; Huang and Tsai, 2024). In this research, the authors studied the effect of psychological empowerment in promoting the mental wellbeing of doctors through the mediating effect of OCB. According to the results of the multiple regression analysis, OCB and psychological empowerment respectively explained 29% and 17% of the variation in doctors' life satisfaction. OCB fully mediated the relationship between psychological empowerment and life satisfaction. According to the study's findings, psychologically empowered medical professionals can encourage behaviors that go above and beyond the call of duty, giving patients greater attention and higher-quality care (Vijayalakshmi, 2023). Hasibuan et al. (2024) investigated the effect of emotional intelligence and job satisfaction on employee performance; the effect of emotional intelligence on employee performance moderated by organizational citizenship behavior; and the effect of job satisfaction on employee performance also moderated by organizational citizenship behavior. The PLS-SEM results reveal a positive and significant relationship between job satisfaction and emotional intelligence and employee performance. Empirical research suggests that augmenting the emotional stability of employees can result in better job performance, which in turn can enhance the overall efficacy of the firm.

Given the importance of OCB and PWB, it is critical to investigate their relationship. Several studies have examined the association between OCB and PWB, with varied degrees of success reported. According to Afsar and Badir (2016), some studies demonstrate a positive correlation between OCB and PWB, while others show a weak or non-significant association. Moreover, little research has been conducted on the relationship between OCB and PWB among workers in the IT industry, which is well known for its demanding job and lengthy workweeks. As a result, the proposed study investigates the relation between OCB, PWB, and EI among workers in the IT-enabled sector employed in Hyderabad city. Emotional intelligence (EI) is an individual's ability to control and express emotions and handle interpersonal relationships meticulously and empathetically (Srivastava, 2013). The study of EI, which is concerned with how humans recognize, understand, control, and perceive emotions, has recently received much attention. Individual differences can potentially impact a number of important outcomes over the course of a person's lifetime.

### 1.1 Research questions

- There exists any relationship between organizational citizenship behavior and psychological wellbeing?
- Is Emotional intelligence is related with psychological wellbeing?
- Is emotional intelligence mediating and moderating on the relationship between organizational citizenship behavior and psychological wellbeing?

## 2 Literature review

During Covid-19 Pandemic employees of several industries in general and Information Technology in particular were asked to work remotely from home or some designated places to mitigate the infection. The remote working employees perceived several psychological challenges during remote working. The challenges include technology issues like increased use and continuous access to internet, smartphone usage, and employee attitude toward the conference applications like Zoom, Microsoft Teams, BlueJeans and other conference applications (Garg et al., 2023). Further, workplace toxicity is another issue which effects the psychological capital (PsyCap). Garg et al. (2023) investigated the relationship between workplace toxicity and psychological capital (PsyCap). It also investigated the moderating role of gratitude in the toxicity–PsyCap link. The authors surveyed 411 subjects working in banking, insurance, IT, automobile and oil and gas companies. The study reported a negative association between toxicity and PsyCap and statistically significant moderating effect of gratitude. The study recommends the institutionalization of a gratitude-based organization to reduce the impact of workplace bullying and uncivil behavior.

Asghar et al. (2022) examined employees' pro-environmental behaviors, such as eco-helping, environmentally friendly civic engagement, and environmentally friendly initiatives to identify the catalysts behind these behaviors. The authors reported that as transformational leaders are inspiring, environmental transformational leadership both directly and indirectly helps to encourage environmental behavior within organizations. Moreover, workers' intentions toward environmental behavior within the organization are stimulated by a strong sense of psychological empowerment and leader-member exchange. In another study, authors reported on a moderated employee mediation study of a Chinese high-tech company examined the opinions of OCB regarding HRM methods, using emotional weariness as the moderator and perceived insider status as the mediator. The association between OCB and perceived insider status is moderated by emotional weariness; employees with low emotional exhaustion levels have a greater relationship with emotional weariness than do those with high emotional exhaustion levels. The association between OCB and perceived HRM practices is somewhat mediated by perceived insider status.

Compulsory citizenship behavior (CCB) has detrimental effects on workers and businesses. Recent studies, however, refute the lack of incentives and positive organizational system evaluation for CCB. Employees who receive resource compensation subsequent to the delivery of CCB will not experience the relative deprivation brought about by hesitant false citizenship behaviors. Furthermore, relative deprivation illustrates the difference between expectations and reality; a small psychological disparity does not significantly impair workers' happiness at work. Qi et al. (2022), in an empirical study, examined the relations between nurses' organizational citizenship behaviors toward their patients and perceived ethical leadership, trust, and psychological wellbeing in the setting of Chinese hospitals. This study showed that nurses' perceptions of ethical leadership are positively correlated with their psychological health and management trust. Management trust is also favorably connected with organizational citizenship practices among nurses. For psychological wellbeing and management trust, the indirect impacts of perceived ethical leadership on OCB were found to be statistically significant. Huang et al. (2021) have examined the reciprocal relationships between PsyCap and its positive affect as well as the crucial role that positive affect plays in the relationships between PsyCap and affective organizational commitment (AOC) and OCB toward that organization (OCBO). These findings are consistent with the theory that positive affect mediates the relationship between OCBO and PsyCap. Additionally, there is some evidence suggesting that PsyCap and positive affect are correlated (Da et al., 2021).

Pradhan et al. (2016) examined the correlation between psychological capital (Psycap) and OCB as well as the potential moderating effect of emotional intelligence on these relationships. The findings indicate a positive relationship between PsyCap and OCB. The main hypothesis of the study, according to which EI modifies the relation between the Psycap and OCB, was also confirmed.

Yang and Zhu (2016) reported that there is a positive correlation between charismatic leadership behavior and increased leadership effectiveness. However, the development of organizational cohesiveness, employees' perceptions of leaders' character, and other psychological empowerment factors are necessary for improving the effectiveness of leadership. Furthermore, the relationship between charismatic leadership behavior and leadership effectiveness can be significantly influenced by both high- and low-emotional intelligence subordinates. However, subordinates with high emotional intelligence find it easier to use charismatic leadership behavior to improve job satisfaction and performance (Yang and Zhu, 2016).

Hsieh et al. (2024) Examined the empowering teachers through emotional intelligence of the principals to exploit the organizational citizenship behavior in Taiwan's elementary schools. The emotional intelligence of principals was assessed by measuring self-awareness, self-management, social awareness, and relationship management dimensions. The teachers' OCB was investigated through interpersonal citizenship performance, organizational citizenship performance dimensions. The results reveal that teachers' OCB is mediated and influenced by principal's emotional intelligence.

The mediating function of OCB in the context of psychological empowerment and life happiness was investigated by Vijayalakshmi (2023). The results of the multiple regression analysis indicated that 29% and 17% of the variation in doctors' life satisfaction was explained by OCB and psychological empowerment, respectively. OCB served as a comprehensive mediator between life satisfaction and psychological empowerment. According to the study's findings, psychologically empowering medical professionals can encourage behaviors that go above and beyond the call of duty, giving patients greater attention and higher-quality care.

Al Zaidi et al. (2023) explored how situational conditions and internal psychological states influence employees' decisions to engage in organizational citizenship activity. Employee perceptions of corporate social responsibility (CSR), as well as all other variables except perceived behavioral control, had a substantial impact on the intention to engage in volunteer pro-environmental behavior. HABs linked with pro-environmental conduct increased the link between intended and actual behavior.

Liu et al. (2024) looked at emotional intelligence (EI) as a potential factor in the relationship between internal CSR and OCBs. The findings show that both environmental and customer-oriented OCBs have a role in mediating the link between internal CSR and WFF. The dual mediation results suggest that EO-OCB, rather than CO-OCB, has a greater mediating influence on the link between internal CSR and WFF.

Hasibuan et al. (2024) investigated the moderating role Organizational Citizenship Behavior in the context of job satisfaction, emotional intelligence and employee performance. According to the study's findings, job satisfaction and emotional intelligence have a positive and significant impact on workers' performance. However, job satisfaction and employee performance do not have any influence on organizational citizenship behavior, and emotional intelligence and employee performance do not influence it.

Santa et al. (2023) evaluated the effect of emotional intelligence on corporate citizenship behavior, transformational and transactional leadership, and, ultimately, operational efficiency. The findings indicate that emotional intelligence has a positive influence on corporate citizenship behavior. However, emotional intelligence has little effect on transformative leadership and only a minor impact on operational performance and transactional leadership. When mediated by organizational citizenship behavior, emotional intelligence has a strong and beneficial impact on operational effectiveness.

## 3 Research problem and research gap

India's IT-enabled economy is dynamic and chaotic, with Hyderabad functioning as a significant IT hub for the country. The industry is distinguished by fierce competition, performance pressure, and excessive work hours that are harmful to employees' wellbeing. This impacts the worker's performance, organizational commitment, and job satisfaction. As a result, it is critical to explore the factors that can boost the emotional intelligence and overall wellbeing of IT-enabled industrial professionals. Whereas OCB may be a contributing factor to employees' wellbeing, emotional intelligence can enhance employees' ability to make decisions. The authors discovered little research on the association between OCB, EI, and PWB among IT-enabled industry employees in Hyderabad Metro, conducting a thorough review of the literature. The authors were unable to locate a single source on OCB, EI, or its relationship to the psychological health of the IT-enabled workforce. There is a limited empirical information on this topic, as well as cross-cultural comparisons demographic variables were not studied. Addressing these study gaps will improve our understanding of OCB and EI at the workplace in India, as well as their relationship with employee PWB.

### 3.1 Objectives

- “To study the association among OCB and EI PWB among IT-enabled sector employees”.
- “To measure the effectiveness of three dimensions of OCB and EI on four dimensions of employee PWB in the IT-enabled sector”.

## 4 Theoretical framework

**Figure 1:**
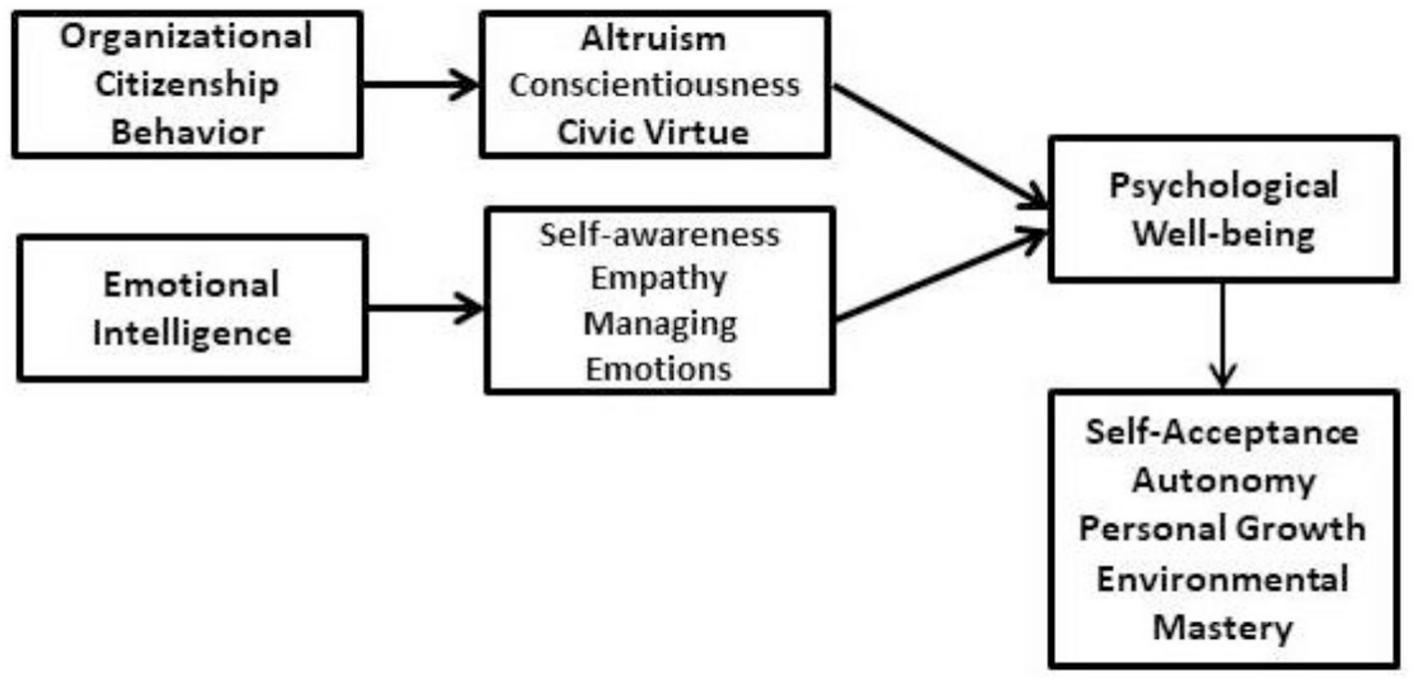
Theoretical framework organizational citizenship behavior, emotional intelligence and psychological wellbeing.

**Figure 2:**
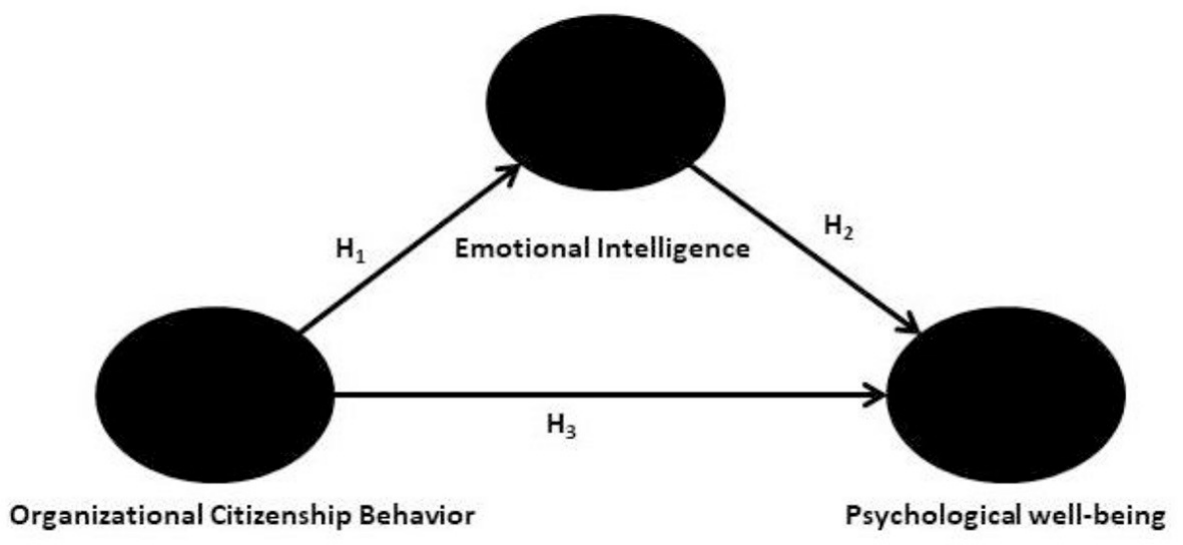
Researchers hypothetical framework.

**Figure 3:**
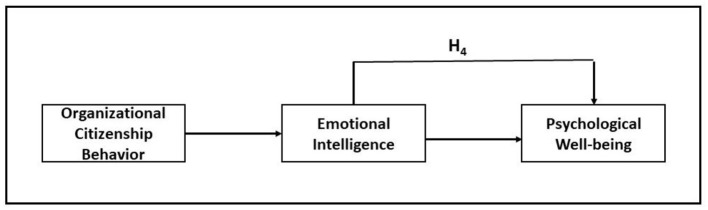
Theoretical model and relationships among the study constructs (adopted from Metselaar et al., 2023).

The theoretical framework (Figure 1) was formulated following the framework of Kumar and Shah (2015) and Ahmadi et al. (2014). The Figure 1 presents that organizational citizenship behavior has three dimensions altruism, conscientiousness, and civic virtue. The three dimensions of OCB impact psychological wellbeing. Emotional intelligence with the three dimensions of self-awareness, empathy, and managing emotions are impacting psychological wellbeing. Psychological wellbeing has four dimensions self-acceptance, autonomy, personal growth, and environmental Mastery. Arpac evaluated the association between organizational citizenship conduct and emotional intelligence in a correlation study involving 114 employees from Istanbul businesses. The author concluded that two aspects of organizational citizenship behaviors—consciousness and altruism—have a favorable link with emotional intelligence. Tofighi et al. (2015) examined the relationship between emotional intelligence and the organizational citizenship behavior of critical emergency nurses in Iran. According to the study, to support critical health care nurses, health care managers should establish dynamic, methodical policies and processes that address emotional intelligence and organizational citizenship behavior. Ghewari and Pawar (2021) studied the mediating role of job satisfaction on organizational citizenship behavior through emotional intelligence to better understand the relationships among these variables. The study's conclusions show that workers with higher EI exhibit more discretionary behavior and are happier in their positions. The results also imply that the association between EI and OCB that has been proposed is somewhat mediated by work satisfaction. Based on these studies, the authors developed hypothetical models and theoretical relationships among the study variables (Figures 2, 3).

### 4.1 Conceptual characteristics of study variables

#### 4.1.1 Organizational citizenship behavior

##### 4.1.1.1 Altruism

This subscale includes items that assess helping behaviors such intentionally supporting others, offering emotional support, and sharing resources.

##### 4.1.1.2 Conscientiousness

This subscale assesses behaviors linked to dependability, reliability, and accountability, including following rules, meeting deadlines, and fulfilling obligations.

##### 4.1.1.3 Civic Virtue

This subscale contains items that assess behaviors associated with an employee's involvement in the organization, such as attending meetings, participating in organizational events, and lobbying for the organization.

#### 4.1.2 Psychological wellbeing

##### 4.1.2.1 Autonomy

The ability to make independent decisions and control one's conduct.

##### 4.1.2.2 Environmental mastery

The sense of control and competence in controlling the environment and reaching personal goals.

##### 4.1.2.3 Personal growth

The sense of continuous development, learning, and progress.

##### 4.1.2.4 Self-acceptance

The ability to accept and appreciate oneself, including strengths and weaknesses.

#### 4.1.3 Emotional intelligence

##### 4.1.3.1 Self-Awareness

Self-awareness is the ability to perceive and understand one's own feelings, which is an important emotional intelligence talent. Being aware of the impact of your behaviors, moods, and emotions on others goes beyond simply recognizing your emotions.

##### 4.1.3.2 Empathy

Another important emotional intelligence trait is empathy, which is the ability to comprehend and see things from the perspective of others. It requires the ability to perceive and comprehend another person's emotional states.

##### 4.1.3.3 Managing emotions

The ability to remain focused and think rationally despite intense emotions. Being able to regulate your own emotional state is critical for accepting responsibility for your actions and can prevent you from making rash judgments that you later regret.

**Figure 4:**
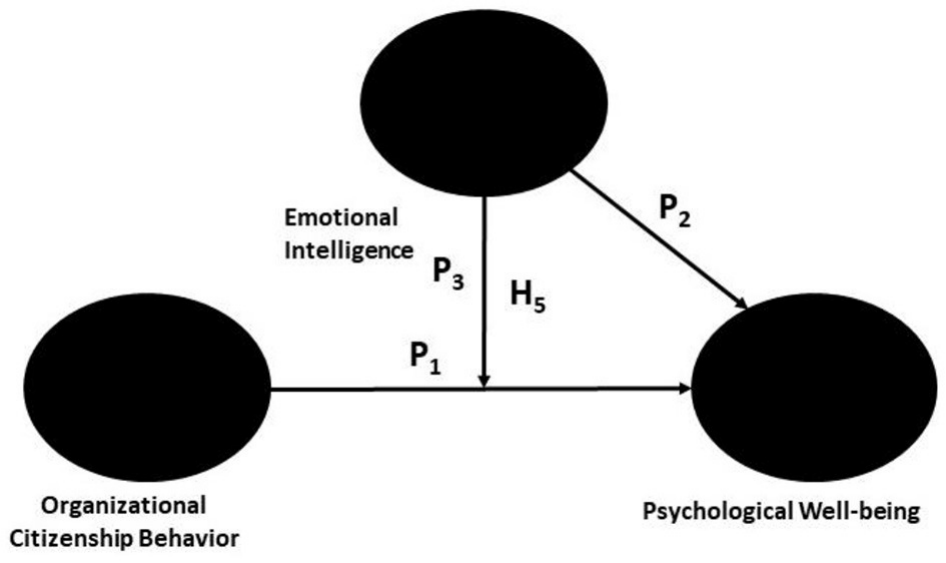
Moderation Model adopted from Hair et al. (2022).

The moderating effect of EI through OCB on PWB was examined following the model of Hair et al. (2022). The moderating effect (P_3_) by an arrow indicates the effect of P_1_ linking OCB and PWB. The moderating impact of the variables was studied via SEM analysis, which revealed that there was a direct relationship between the moderator (EI) and the dependent construct PWB (P_2_) (Figure 4).

## 5 Hypotheses

The following hypotheses were proposed after the study's objectives were developed and the research gaps that were identified were considered. Figure 2 presents the researcher's theoretical framework.

H_1_: Organizational citizenship behavior has strong ramifications for the emotional intelligence of IT-enabled industry employees.
H_2_: Emotional intelligence has a statistically significant influence on the psychological wellbeing of IT-enabled industry employees.
H_3_: Organizational citizenship behavior has a statistically significant impact on the psychological wellbeing of IT-enabled industry employees.
H_4_: Emotional intelligence mediates the relationship between psychological wellbeing and organizational citizenship behavior of IT-enabled industry employees.
H_5_: Emotional intelligence moderates the relationship between psychological wellbeing and organizational citizenship behavior of IT-enabled industry employees.

## 6 Methodology

Most of the previous studies used partial least squares-structural equational modeling and studied the constructs separately like organizational citizenship behavior vs. psychological wellbeing, and emotional intelligence vs. psychological wellbeing. Most of the other studies used two more constructs such employee performance, job satisfaction along with the emotional intelligence and organizational citizenship behavior. The past studies not modeled the constructs organizational citizenship behavior, emotional intelligence and psychological-wellbeing. This study used three reflective constructs and used IBM AMOS veer 28 to model the study and test the hypotheses. The empirical research was conducted using three standardized questionnaires—a 24-item OCB scale by Organ (1994), an 18-item PWB scale developed by Ryff and Keyes (1995), and an emotional intelligence 27-item scale developed by Srivastava et al. (2011). The scales were adjusted to fit the present study, and a 20-item scale was constructed to represent all three reflective constructs. The OCB dimension has 5 items, EI has 7 items and PWB has 8 items. The assessed Cronbach's alpha was 0.83, and the split half (odd-even) reliability was 0.85. These findings indicate that the questionnaire was reliable and consistent. The data were analyzed via SEM analysis to assess the effect of OCB and EI on the psychological wellbeing of IT sector-enabled employees. A survey instrument was developed, published in Google form, and shared online through a variety of platforms, including LinkedIn, WhatsApp, and emails, to gather the data. Five hundred workers in the information technology sector were sent a link to the questionnaire. A total of 300 valid responses were taken into account for the structural equation modeling data analysis.

### 6.1 Sample size estimation

The IT-enabled sector population is unknown, and the minimum sample size required for the unknown population according to Cochran's (1977) formula is 384. However, this study used 300 valid responses for data analysis, using the formula for a minimum sample size of 50+5x, where x represents the number of questions (Gaskin et al., 2023). The present study has 40 questions, and 250 is the needed sample size per this formula (Gaskin et al., 2023); the sample size of 300 for this research is greater than the estimated sample size of 250.

### 6.2 Data collection

Two measures of organizational citizenship behavior and emotional intelligence were administered: a five-point Likert-type scale ranging from “Strongly agree 5” to “Strongly disagree 1”. A seven-point scale (Ryff and Keyes, 1995) was used to assess the PWB of IT-enabled employees in Hyderabad. To facilitate easy computation, the seven-point PWB measure responses were converted using linear transformation techniques to a five-point Likert-type scale. (IBM SPSS 28; Prasad et al., 2020). The demographic characteristics of study sample presented in Table 1.

**Table 1:** Demographic characteristics of the study sample.

**Item**	**Frequency**	**Percent**
**Gender**		
Male	156	52.00
Female	144	48.00
**Age group**		
20–30	81	27.00
31–40	74	24.67
41 Plus	145	48.33
**Marital status**		
Married	156	52.00
Un Married	144	48.00
**Education**		
SSC	34	11.33
Graduate	119	39.67
Post-Graduate	126	42.00
Other	21	7.00
**Children**		
Yes	120	40.00
No	180	60.00
**Experience (years)**		
1–5	39	13.00
6–10	114	38.00
11–20	111	37.00
>20 Years	36	12.00

### 6.3 Reliability and internal consistency

The factor analysis results yielded three components and the outer loadings are presented in the Table 2. The Cronbach's alpha reliability statistic was used to evaluate the questionnaire's reliability and internal consistency. The results, as shown in Table 3 and the Spearman-Brown Prophecy, show the internal consistency and reliability of the instrument.

**Table 2:** Items for study variables and outer loadings.

**Emotional intelligence**	**Outer loading**
“EI1 - I realize immediately when I lose my temper”	0.86
“EI2 - I know when I am happy”	0.89
“EI3 - I usually recognize when I am stressed”	0.79
“EI4 - I can reframe bad situations quickly”	0.81
“M5 - Others can rarely tell what kind of mood I am in”	0.79
“EI6 - I am always able to see things from the other person's viewpoint”	0.87
“EI7 -I am excellent at empathizing with someone else's problem”	0.68
**Organizational citizenship behavior**	
“OCB1 -I help others who have heavy workloads”	0.72
“OCB2 - I am always ready to lend a helping hand to those around me”	0.92
“OCB3 - I do not tend to make “Mountains out of molehills”.	0.73
“OCB4- I attend functions that are not required by help the company image”	0.88
“OCB5 - I read and keep up with organization announcements, memos and so on”	0.70
**Psychological wellbeing**	
“PWB1 – 1 I like most parts of my personality.”	0.76
“PWB2- 2 When I look at the story of my life, I am pleased with how things have turned out thus far.”	0.73
“PWB3 - I tend to be influenced by people with strong opinions”	0.74
“PWB4 - I have confidence in my own opinions, even if they are different from the way most other people think”	0.83
“PWB5 -The demands of everyday life often get me down.”	0.84
“PWB6 - In general, I feel I am in charge of the situation in which I live.”	0.84
“PWB7 - For me, life has been a continuous process of learning, changing, and growth.”	0.80
“PWB8 -I think it is important to have new experiences that challenge how I think about myself and the world.”	0.79

**Table 3:** Reliability and Convergent Validity of the constructs.

**Factor**	**Cronbach's alpha**	**Composite reliability**	**Average variance extracted/convergent validity**
Psychological wellbeing	0.81	0.930	0.626
Emotional intelligence	0.72	0.933	0.669
Organizational citizenship behavior	0.88	0.893	0.628

Source: Primary data processed.

## 7 Data analysis

The factor analysis was conducted using IBM-SPSS version 28. To test the hypotheses, the data were analyzed via SEM (IBM AMOS 28). The authors examined both the inner and exterior measurement models. There are 20 indicators across 3 reflective constructs in the current study. In a number of investigations and social sciences studies using small and large sample sizes and nonnormal and normal data, researchers have used the IBM-AMOS model to quantify absolute path coefficients (Hair et al., 2013).

## 8 Results and discussion

The results of the SEM, structural model, mediation and moderation analyses are presented and described, and the hypotheses are tested. This empirical research has 10 reflective latent constructs. To establish the reflective measurement, the validity and reliability being assessed for suitability for additional research (Hair et al., 2011).

## 9 Measurement model

**Figure 5:**
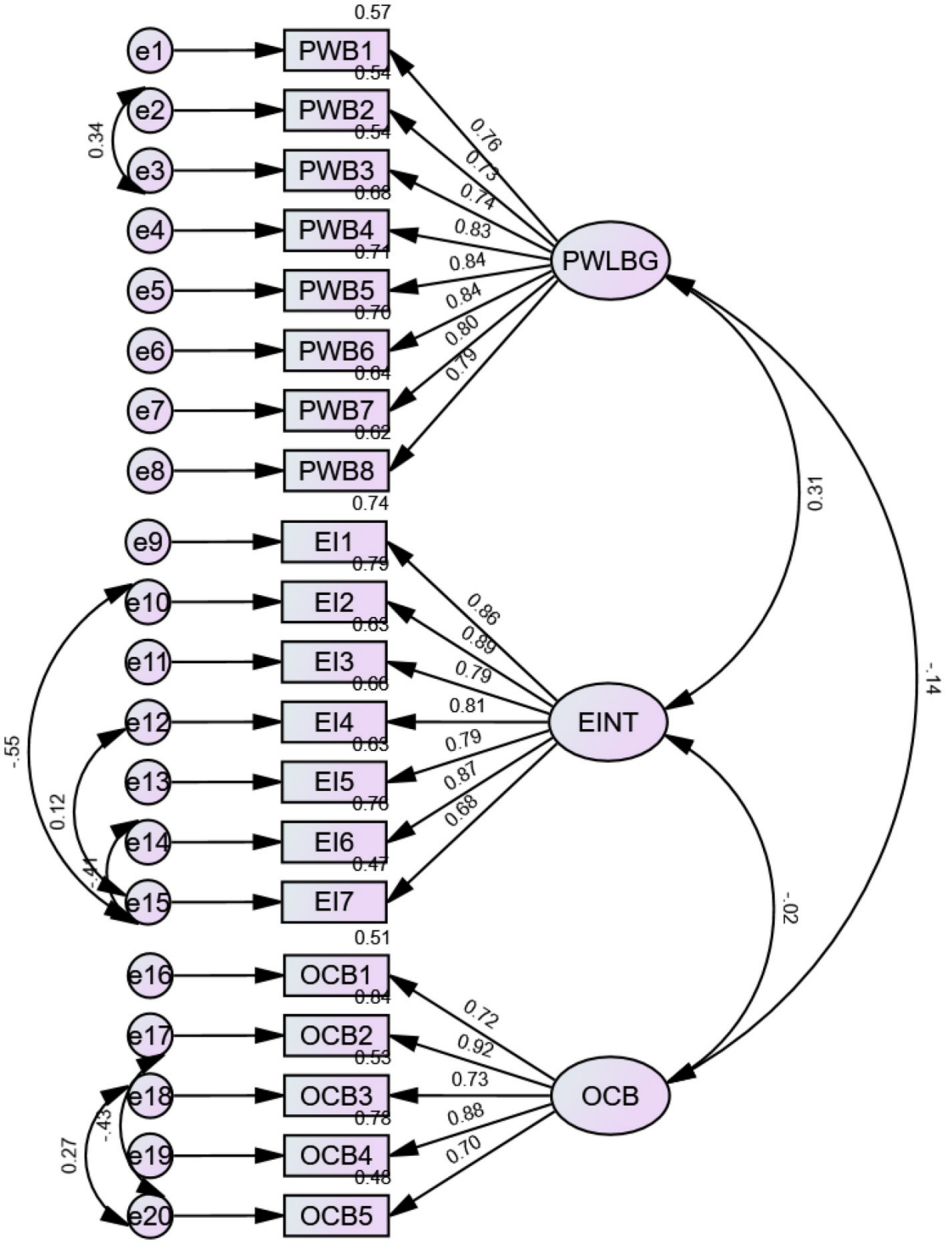
Measurement model. OCB, organizational citizenship behavior; EI, emotional intelligence; PWB, Psychological wellbeing.

The confirmatory factory analysis (CFA) was computed using AMOS to test the measurement model. As part of the CFA, factor loadings were assessed for each item (Figure 5). The model-fit measures were used to assess the model's overall goodness of fit (CMIN/df, GFI, CFI, TLI, SRMR, and RMSEA); all the values were within their respective recommended and common acceptance levels (Bentler and Bonett, 1980; Hu and Bentler, 1998; Ullman, 2001). The three-factor model (organizational citizenship behavior, psycological wellbeing and emotional intelligence) fit the data well (Table 4). The factor loading values (Kline, 2015) are excellent, acceptable, and nonnegative, and all are greater than 0.5, with an average factor loading >0.7 for all three constructs; additionally, the model has an excellent fit, as presented in Table 4 (Byrne, 2013). The measurement model is presented in Figure 5.

**Table 4:** Model fit statistics.

**Item**	**Estimate**	**Range**	**Reference**
CMIN	1,075.918		
DF	692.000		
Relative Chi-Square (CMIN/DF)	2.788	< 3	Kline, 2011
Comparative Fit Index (CFI)	0.953	>0.90	Bentler and Bonett, 1980
Incremental Fit Index (IFI)	0.937	>0.90	Bollen and Lennox, 1991
Tucker Lewes Index	0.921	“>0.90”	Tucker and Lewis, 1973”
Normed Fit Index	0.923	“>0.90”	Bentler and Bonett, 1980
Root Mean Square Error of Approximation (RMSEA)	0.054	0.5 or less	MacCallum et al., 1993
Standardized Root Mean Square Residual (SRMR)	0.043	< 0.05	MacCallum et al., 1993
PClose	0.019	>0.05	James et al., 2009

The construct reliability was assessed using Cronbach's alpha and composite reliability. Cronbach's alpha for each construct in the study was measured above the recommended value of >0.70 (Nunnally and Bernstein, 1994). The composite reliabilities ranged from 0.917 to 0.952 above the recommended and benchmark values of 0.70 (Hair et al., 2011). Therefore, construct reliability was established (Table 3).

The convergent validity of the scale items was estimated using the average variance extracted (AVE) (Fornell and Larcker, 1981). The AVE values were above the threshold of 0.50 (Fornell and Larcker, 1981. Hence, the scales used in this empirical study have convergent values (Table 3).

Discriminant validity illustrates how a specific construct varies from other constructs and explains how closely correlated the measures should be (Anderson and Gerbing, 1988). Discriminant validity was assessed in the present study using the Fornell–Larcker criterion and the heterotrait–monotrait (HTMT) ratio. According to the Fornell and Larcker criterion, discriminant validity is established when the square root of the AVE for a construct is greater than its correlation with the other constructs in the study. However, the Fornell and Larcker criterion has recently been criticized, and a new method for assessing discriminant validity, the HTMT ratio, has been increasingly utilized. In the present study, discriminant validity was not established using the Fornell and Larcker criterion. However, when assessed using the HTMT ratio, all ratios were less than the required limit of 0.85 (Henseler et al., 2015). Therefore, discriminant validity was established (Tables 5, 6).

**Table 5:** Discriminant validity.

	**Psychological wellbeing**	**Emotional intelligence**	**Organizational citizenship behavior**
Psychological wellbeing	**0.791**		
Emotional intelligence	0.314^***^	**0.818**	
Organizational citizenship behavior	−0.138^*^	−0.018	**0.792**

^*^significant at < 0.05 level; ^***^ significant at < 0.001 level. Values in bold are the square root of the average variance extracted by a construct.

**Table 6:** Heterotrait-monotrait analysis.

	**Psychological wellbeing**	**Emotional intelligence**	**Organizational citizenship behavior**
Psychological wellbeing			
Emotional intelligence	0.292		
Organizational citizenship behavior	0.129	0.005	

## 10 Structural model

**Figure 6:**
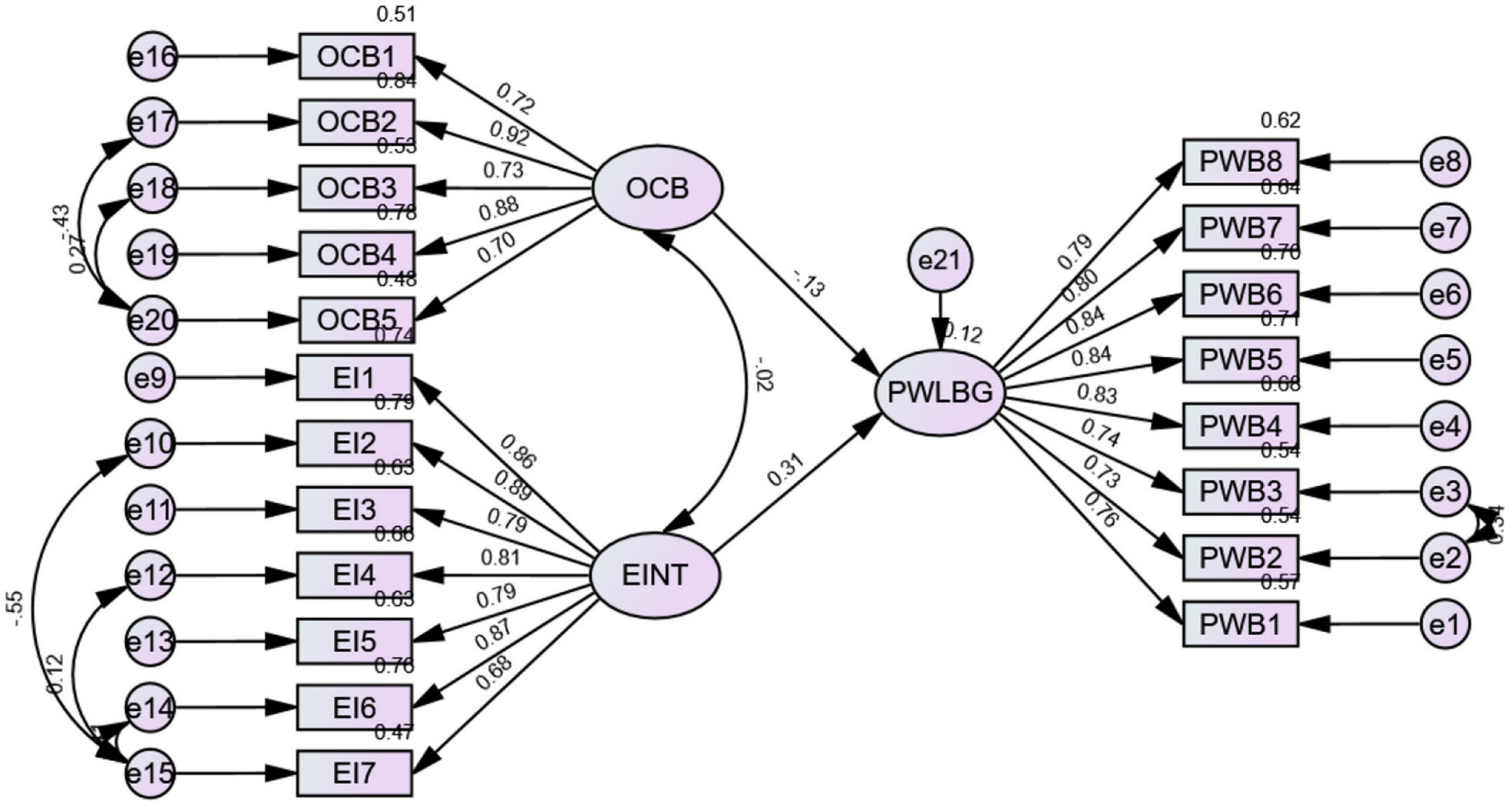
Structure model with relationships. OCB, organizational citizenship behavior; EI, emotional intelligence; PWB, psychological wellbeing.

A structural equation model generated through AMOS was used to test the relationships. A good fitting model is accepted if the CMIN/df is < 5, the GFI is < 0.90 (Hair et al., 2011), the Tucker and Lewis indices are >0.90 (1973), and the confirmatory fit index (CFI) (Bentler, 1990) is >0.90 (Hair et al., 2011). In addition, an adequate-fitting model was accepted if the AMOS-computed value of the standardized root mean square residual (RMR) was < 0.05 and the root mean square error approximation (RMSEA) ranged between 0.05 and 0.08 (Hair et al., 2011). CMIN/DF 2.788 CFI 0.935, IFI 0.937, TLI 0.921, NFI 0.923, RMSEA 0.054, SRMR 0.043 and PClose 0.092. The indices indicated in Table 4 fall within the acceptable range (Figure 6).

The squared multiple correlation was 0.12 for psychological Wellbeing, which indicates that 12% of the variance in psychological Wellbeing is accounted for by Organizational Citizenship Behavior and Emotional Intelligence.

### 10.1 Common method bias

Common method bias, or CMB, is the inflation or depletion of the true correlation between the study's observable variables (Kock, 2015). Artificial inflation of covariance is achievable because respondents usually answer questions involving both independent and dependent variables simultaneously. Using the common method latent factor and Harman's single-factor test, this study evaluated common method bias.

#### 10.1.1 Harman's single factor test

Confirmatory factor analysis was used to evaluate the model fit after the researchers loaded all the indications onto a single factor. After verification, the model fit was not appropriate, ruling out common approach bias.

#### 10.1.2 Latent common method factor

A latent construct with a direct relationship to each of the construct's model indicators was employed by the writers. A latent construct known as the common method was sketched. Subsequently, the model contained a direct correlation between each indicator in the model and the latent construct of the unobserved common technique. A path from the common method construct to each indicator in the model is drawn, and then, all the relationships from the method factor are constrained to be the same to determine whether there is a common impact among all the items/indicators. The model was run using the latent common method variable, which is directly related to all of the variables; the chi-square value of this CFA model was noted. The value of 1,080.102 is the observed chi-square value, and there are 697 degrees of freedom. With 698 degrees of freedom, the basic model's chi-square without a latent factor is 1,075.918. The chi-square difference of 4.184 suggested the presence of common method bias. However, since the CMB is so low and has little bearing on the study's findings, it is not a significant problem in this work (Tables 7, 8).

**Table 7:** CMIN (without latent common method).

**Model**	**NPAR**	**CMIN**	**DF**	**P**	**CMIN/DF”**
Default model	90	1,075.918	698	0.024	1.555
Saturated model	314	0.000	0		
Independence model	54	8,361.918	750	0.000	11.1493

**Table 8:** CMIN (with latent common method).

**Model**	**NPAR**	**CMIN**	**DF**	**P**	**CMIN/DF**
Default model	90	1,630.98	697	0.039	2.340
Saturated model	275	0.000	0		
Independence model	54	8,361.918	750	0.000	11.149

## 11 Testing of hypotheses

Table 9 shows that the path coefficient of OCB is statistically significant, influencing emotional intelligence (ß = 0.328; *p* < 0.001) and explaining 33% of the variance; moreover, when OCB increases by 1 unit, emotional intelligence increases by 0.328 units (Table 9). Therefore, hypothesis **“H**_**1**_**: Organizational citizenship behavior has high ramifications for the EI of IT-enabled industry employees” is supported**.

**Table 9:** Estimates of structural equation modeling (hypothesis testing).

**Hypotheses of study**	**Beta/path coefficient**	***t*-statistic**	***p* value**	**Decision**
H_1_: OCB → EI	0.328	4.065	< 0.001	Supported
H_2_:EI → PWB	0.601	4.165	< 0.001	Supported
H_3_: OCB → PWB	0.264	4.161	< 0.001	Supported
H_4_: EI mediates PWB	0.264	4.077	< 0.001	Partially Supported

Source: Primary data processed.

Emotional intelligence significantly and positively influences the psychological wellbeing of employees in IT-enabled industries (ß2 = 0.601; *p* < 0.01). When emotional intelligence is increased by 1 unit, the psychological wellbeing of employees increases by 0.601 (Table 9) units, which explains 60% of the variance; therefore, “H_2_: Emotional intelligence has a statistically significant influence on the psychological wellbeing of IT-enabled industry employees”.

Furthermore, organizational citizenship behavior significantly influences emotional intelligence (ß3 = 0.264; *p* < 0.001), explaining 26% of the variance in psychological wellbeing (Figure 6). This finding supports H_3_: Organizational citizenship behavior has a statistically significant impact on the psychological wellbeing of IT-enabled industry employees.

## 12 Mediation analysis

A mediator is a third variable that acts as an intermediary in the indirect path of influence between two constructs. At this point, the influence of the two conceptions is affected by the third variable (Hair et al., 2011). An intervening variable is another name for a mediating variable. To determine the impact of mediating variables, direct, indirect, and total impacts must be evaluated.

**Figure 7:**
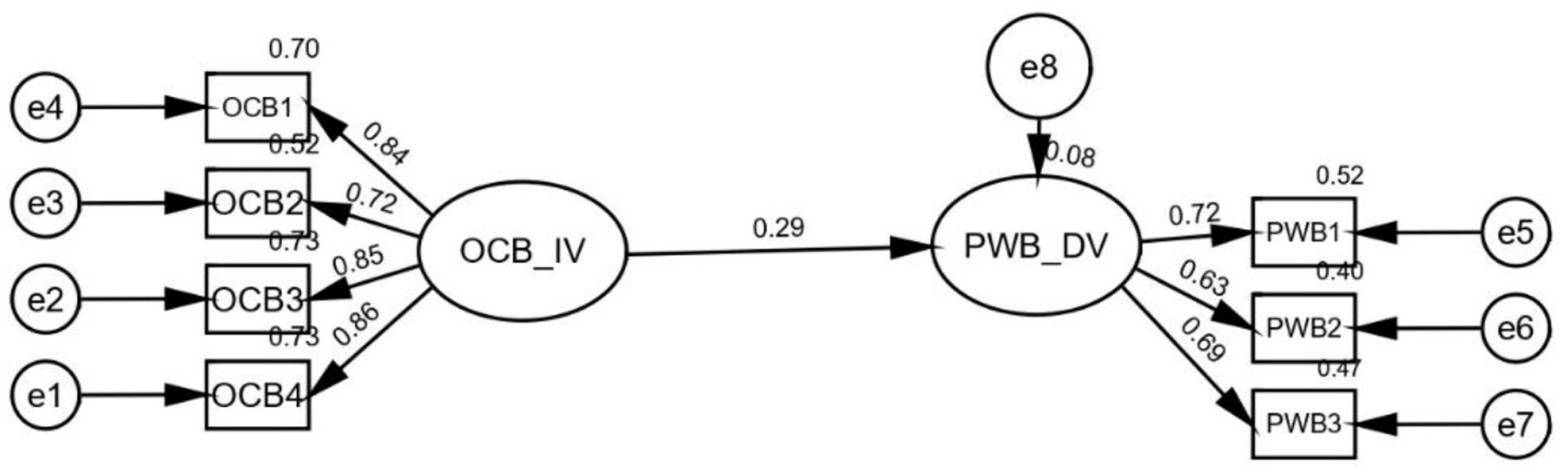
Direct relationship among independent variable organizational citizenship behavior with dependent variable psychological wellbeing in the absence of mediator.

The direct impact of the independent construct (OCB) on the dependent construct (PWB) in the absence of a mediator was examined to evaluate the mediating effect. Additional mediation analysis was performed if the outcome was deemed to be significant (Hair et al., 2016). Afterwards, the process of bootstrapping is employed to evaluate the confidence intervals (Cheung and Lau, 2008; Mahfud et al., 2020). Figure 7 shows that organizational citizenship conduct has a significant and beneficial effect on the psychological wellbeing of IT sector employees when no mediator is present. Additional mediation analysis was performed because the estimates were statistically significant (ß = 0.29; *p* < 0.001).

## 13 Mediation analysis results

To compare the indirect effects in the model and measure the mediating effect of emotional intelligence on the psychological wellbeing of IT personnel, the author of the study adhered to Preacher and Hayes (2008) methodology. This study examined the mediating variable's indirect effect. Huang et al. (2018) studies the mediating effect of altruistic behavior and confirms that altruistic behavior has positive mediating effects on subjective wellbeing. Dev and Rahman (2016) reported the positive mediating effects of emotional intelligence on physical activity and mental health among Malaysian students. Lanciano et al. (2012) reported the mediating role of emotional intelligence and concluded that emotional intelligence is positively related to dysfunctional rumination.

**Figure 8:**
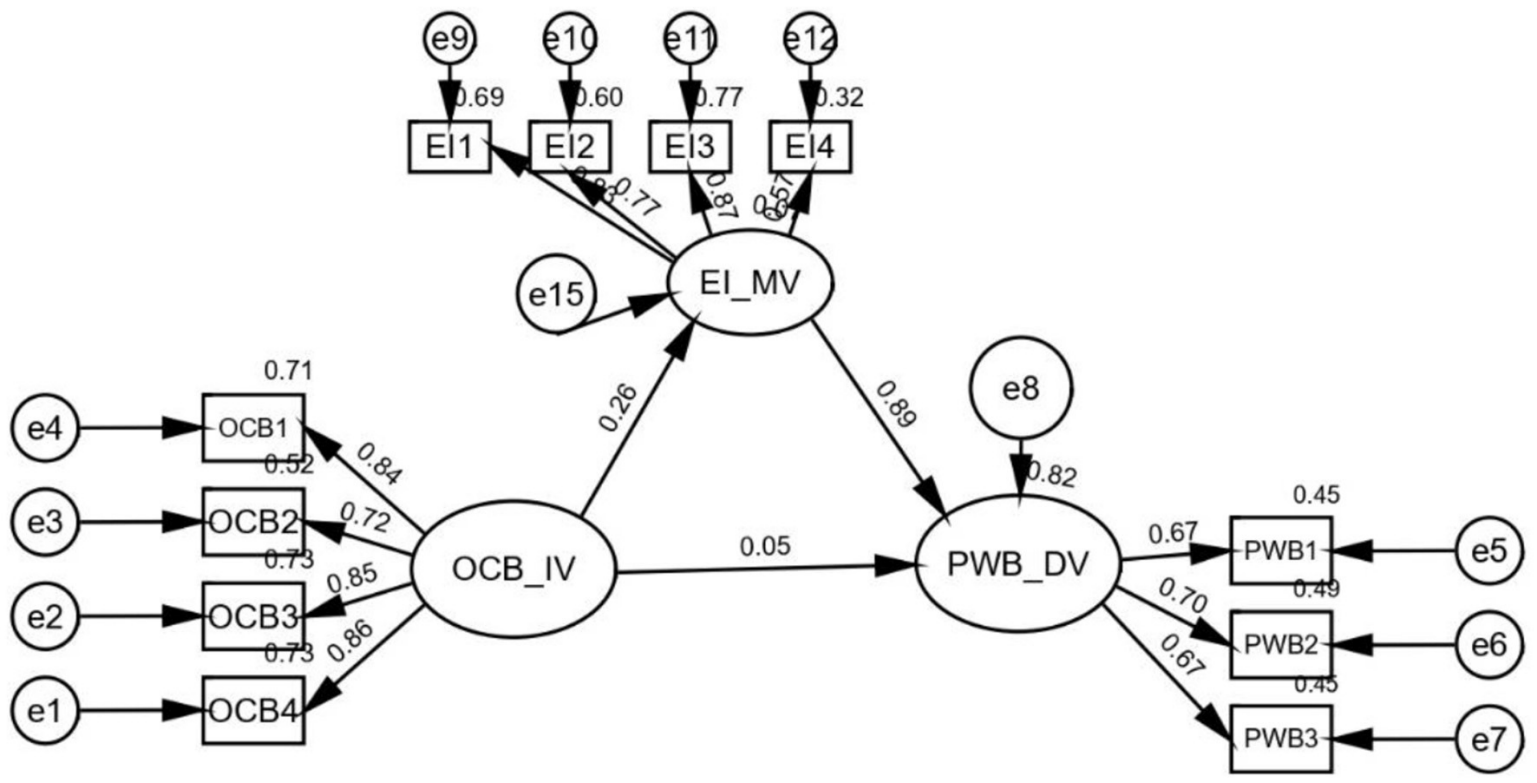
Mediation analysis. OCB, organizational citizenship behavior; EI, Emotional intelligence; PWB, Psychological wellbeing.

The study evaluated how emotional intelligence mediated the association between psychological wellbeing and organizational citizenship behavior. The results are statistically significant (b = 0.264, t = 4.073, *p* ≤ 0.001), further supporting H_4_: Emotional intelligence mediates psychological wellbeing. Furthermore, regarding the direct effect of OCB on psychological wellbeing, it was discovered that emotional intelligence was significantly related to the presence of the mediator (b = 0.197; *p* = 0.000). Hence, emotional intelligence partially mediated the relationship between organizational citizenship behavior and psychological wellbeing (Table 10, Figure 8). Thus, “**H**_**4**_**: Emotional intelligence has a mediating effect on the psychological wellbeing of IT employees”**.

**Table 10:** Mediation analysis summary.

**Relationship**	**Direct effect**	**Indirect effect**	**Confidence Interval**		***P* value**	**Conclusions**
			**Lower bound**	**Upper bound**		
Organizational citizenship behavior **→** Emotional intelligence **→**Psychological wellbeing	0.264 (0.000)	0.197 (0.000)	0.102	0.308	0.000	Partial mediation

Is there any significance of indirect paths?

- The indirect can be calculated multiplying the path coefficients between OCB → EI (0.328) and EI → PWB (0.601).
  The total indirect effect of 0.197 (Table 8) is the product of a1^*^b1 =0.328^*^0.601= 0.197.
  Organizational citizenship behavior → Emotional intelligence= 0.328: Emotional Intelligence → Psychological Wellbeing = 0.601.

### 13.1 Moderation analysis

To assess the influence of the moderator variable Emotional Intelligence on the direct influence of OCB on the PWB of IT-enabled industry employees, moderation analysis was carried out. According to our moderation analysis, the moderator variable emotional intelligence interacts with organizational citizenship behavior as an independent variable. The authors applied the “interaction term” method of moderation to form the product term of the moderator variable and the independent variable. Another reason for carrying out the “interaction term” method of moderation as the moderator variable emotional intelligence is through the use of a continuous variable. The authors used the path model with composite variables. We have assessed how the interaction between OCB and EI influences the PWB of IT-enabled employees.

The problem/issue of high collinearity (Frazier et al., 2004) with the product term of the moderator and independent variable was overcome by mean centering the variables of the study. However, the results will be similar even when moderation analysis is performed without mean centering the variables on raw data (Echambadi and Hess, 2007; Hayes, 2018). The advantage of the mean of catering to the data are that it will circumnavigate potential collinearity issues, and interpretation of the results is easier. Therefore, we have mean centered the data before analyzing them (Dawson, 2014).

### 13.2 Moderation analysis results

The relationship between OCB and PWB moderated with emotional intelligence. To study this phenomenon, we examined the moderating role of emotional intelligence (EI) on the relationship between OCB and the PWB of IT-enabled employees. The results reveal a positive and statistically significant moderating impact of emotional intelligence on the relationship between organizational citizenship behavior and psychological wellbeing (b = 0.095, t = 2.195, *p* < 0.001; Table 11).

**Table 11:** Summary of the moderation analysis.

**Relationship**	**Beta**	**CR**	***P* value**
EI **→**PWB	0.588	17.253	< 0.001
OCB^*^EI **→**PWB	0.095	2.195	< 0.001

**Figure 9:**
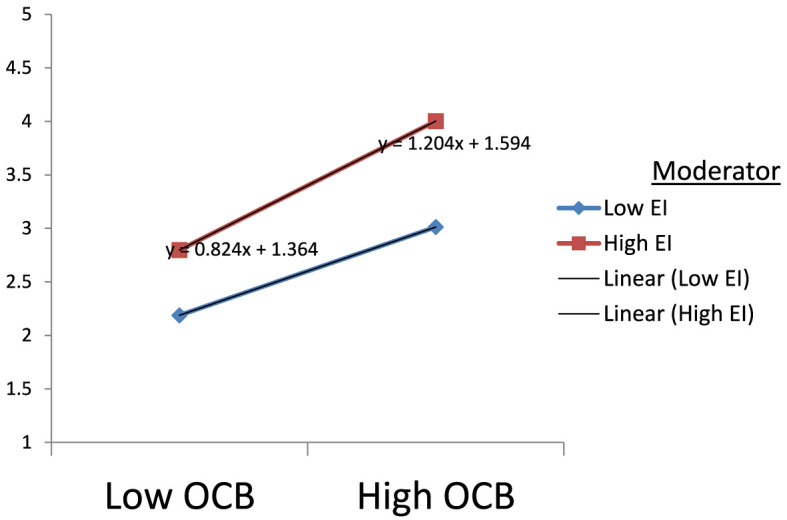
Emotional Intelligence strengthens the positive relationship between organizational citizenship behavior and psychological wellbeing.

To better understand the nature of the moderating effects of emotional intelligence on psychological wellbeing through organizational citizenship behavior, a slope analysis was carried out (Figure 9). Figure 9 clearly shows that at a high level of Emotional Intelligence, the impact of Organizational Citizenship Behavior on the Psychological Wellbeing of IT-enabled industry employees is much greater than that at a low level of Emotional Intelligence. When the level of employee EI increases, the strength of the relationship between OCB and PWB increases.

## 14 Discussion

To dissect the relationship between OCB, EI and PWB among IT-enabled industry employees around Hyderabad, this study was carried out. This study also aimed to fill this research gap using three constructs, as past studies have been limited to only two variables, OCB and PWB in general and EI and PSB in particular. The authors surveyed employees working in an IT-enabled industry using a questionnaire with 40 items to measure the three constructs, OCB and EI, and 4 constructs, PWB. This empirical research examines the association between OCB and EI and between EI and PWB. The organizational citizenship behavior and emotional intelligence variables are positively associated with psychological wellbeing

The findings indicate that EI and OCB are statistically significant, and among IT-enabled employees, there is a substantial correlation between these factors and PWB characteristics. Our findings are consistent with these findings. Huang et al. (2021) Organizational citizenship behavior: mediation of trust and psychological wellbeing A similar study was conducted by Alfonso et al. (2016) to evaluate the mediating function of work-life quality in relation to emotional intelligence and organizational citizenship behavior. Pradhan et al. (2016) studied the moderating role of emotional intelligence on psychological capital and reported similar results.

The findings of this study have several important implications for organizations in the IT sector and can be used to develop strategies for promoting OCB and EI among employees. Kang et al. (2020) dissected the structural relationships between PWB and OCB in the context of hotel employees and reported positive effects of OCB on hotel employees.

Employee OCB has the potential to improve PWB, boost performance, and increase job satisfaction. Additionally, the study revealed that employees with high EI are more likely to have improved PWB and performance. Additionally, this study highlighted the importance of creating a positive working environment with organizational citizenship behavior that fosters emotional intelligence, as it directly impacts the psychological wellbeing of IT employees. Jain (2012) reported the association between emotional intelligence and organizational citizenship behavior with the moderating effect of impression management. Emotional intelligence and organizational citizenship behavior are positively moderated by impression management.

According to the report, as emotional intelligence can result in better PWB, job satisfaction, and higher employee performance, the IT business should support and develop emotional intelligence among its workers.

## 15 Conclusion

This research has provided evidence of the positive association between OCB, EI and PWB among IT-enabled industry employees. It is evident from the results that a positive organizational culture enhances the emotional intelligence that encourages and rewards such behaviors. The study has also identified specific components of OCB, and EI is essential for enhancing PWB. Altruism, diligence, civic virtue, and corporate citizenship conduct, as well as empathy, self-awareness, and emotional intelligence management, are crucial elements for improving PWB. The research and practical implications of these findings are examined, highlighting the potential advantages of OCB and EI promotion for improving employee wellbeing and organizational success. Overall, this study adds significantly to the body of knowledge on OCB and EI and how they affect worker wellbeing in the context of India's IT industry.

### 15.1 Research implications

The findings of this study have several important implications for organizations in the IT sector and can be used to develop strategies for promoting OCB and EI among employees. Kang et al. (2020) dissected the structural relationships between PWB and OCB in the context of hotel employees and reported positive effects of OCB on hotel employees. The limitations are the data were collected from the Information Technology employees of Hyderabad Metro. There are some subjectivity and cultural issues which were elaborated at the end

### 15.2 Limitations

The Hyderabad Metro area's IT-enabled industry made up the entire study sample. Nonetheless, a larger sample size could be used to generalize the results. Although the study focused on self-reported measures of emotional intelligence, organizational citizenship behavior, and psychological wellbeing, biases such as response bias and social desirability bias may have affected the findings. The researchers used reversal questions, checks, and variance measurements to prevent self-reported bias when participants provided answers that were not totally accurate or truthful. The reliability statistics suggest that the sample size of 300 individuals is considerable and may be representative of the population; however, this may also limit how broadly the findings may be applied. Other study limitations include the absence of a control group, cultural considerations, and problems with the cross-sectional design. The other limitations are as follows:

**Subjectivity:** Subjective notions such as OCB, EI, and psychological wellbeing can be challenging to measure with precision. It can be challenging to compare and analyze data since various people may have different meanings and interpretations of these ideas.
**Culture:** Cultural elements, such as expectations, conventions, and values, may have an impact on psychological health, OCB, and emotional intelligence. It may be challenging to generalize research from one culture to another as a result.
**Self-report bias:** Self-report measures are used in many studies on OCB, EI, and psychological wellbeing, although they might be biased. Inaccurate outcomes may arise from people reporting their own actions or feelings inaccurately or from social desirability bias.
**Multidimensionality:** A variety of circumstances can impact the complicated, multifaceted concepts of OCB, EI, and psychological wellbeing. This approach can make it challenging to separate out the many variables and pinpoint precise causal linkages.

## Data availability statement

The original contributions presented in the study are included in the article/Supplementary material, further inquiries can be directed to the corresponding author.

## Ethics statement

Ethical review and approval was not required for the study on human participants in accordance with the local legislation and institutional requirements. Written informed consent from the [patients/ participants OR patients/participants legal guardian/next of kin] was not required to participate in this study in accordance with the national legislation and the institutional requirements.

## Author contributions

KP: Conceptualization, Data curation, Methodology, Writing – original draft, Writing – review & editing, Formal analysis. RV: Conceptualization, Project administration, Writing – original draft, Writing – review & editing. VS: Data curation, Methodology, Writing – review & editing. SS: Conceptualization, Formal analysis, Methodology, Supervision, Resources, Software, Writing – review & editing. KD: Conceptualization, Supervision, Project administration, Resources, Visualization, Writing – original draft.
